# Loss of Myeloid BMPR1a Alters Differentiation and Reduces Mouse Prostate Cancer Growth

**DOI:** 10.3389/fonc.2020.00357

**Published:** 2020-04-07

**Authors:** Claire L. Ihle, Desiree M. Straign, Meredith D. Provera, Sergey V. Novitskiy, Philip Owens

**Affiliations:** ^1^Cancer Biology Graduate Program, University of Colorado Anschutz Medical Campus, Aurora, CO, United States; ^2^Department of Pathology, University of Colorado Anschutz Medical Campus, Aurora, CO, United States; ^3^Department of Pathology, Microbiology and Immunology, Vanderbilt University Medical Center, Nashville, TN, United States; ^4^Department of Veterans Affairs, Research Service, Eastern Colorado Health Care System, Aurora, CO, United States

**Keywords:** BMP, tumor microenvironment, myeloid cells, prostate cancer, macrophage polarization

## Abstract

The Bone Morphogenetic Protein (BMP) pathway is a member of the TGFβ signaling family and has complex roles in cancer. BMP signaling is rarely mutated and can be frequently overexpressed in many human cancers. The dichotomous role of BMPs as both tumor promoters and suppressors appears to be largely context based in both the cancer cell and the surrounding microenvironment. Myeloid cells including macrophages and neutrophils have been shown to be tumor promoting when stimulated from BMPs. We found that conditional deletion of BMPR1a in myeloid cells (LysMCre) restricts tumor progression in a syngeneic mouse prostate cancer model. Specific changes occurred in myeloid cells both in tumor bearing mice and tumor naïve mice throughout multiple tissues. We profiled myeloid subsets in the bone marrow, spleen and primary tumor and found myeloid BMPR1a loss altered the differentiation and lineage capability of distinct populations by histologic, flow cytometry and high dimensional mass cytometry analysis. We further confirmed the requirement for BMP signaling with pharmacologic inhibition of THP-1 and Raw264.7 activated into M2 macrophages with the BMP inhibitor DMH1. M2 polarized primary bone marrow derived cells from LysMCre BMPR1a knockout mice indicated a distinct requirement for BMP signaling in myeloid cells during M2 activation. These results indicate a unique necessity for BMP signaling in myeloid cells during tumor progression.

## Introduction

The bone morphogenetic proteins (BMPs) are members of the transforming growth factor-β (TGF-β) super-family and exhibit diverse roles during development and tissue homeostasis. BMPs bind to two types of serine/threonine kinase transmembrane receptors, type I and type II. Type I receptors consist of seven different activin receptor-like kinases (ALK) numbered 1 through 7. ALK-3, also known as BMPR1a, acts as a type I receptor to activate downstream canonical Smad signaling in cells after BMP ligand binding. Smad dependent BMP signaling in multiple cell lineages drives inhibitor of differentiation-1 (*ID1*) gene expression. Induction of *ID1* suggests BMPs are regulators of differentiation in a variety of cell types (1).

BMPs were first discovered for their role in the formation of bone (2). BMPs are involved in differentiation of mesenchymal stem cells into bone forming osteoblasts and cartilage forming chondroblasts to participate in skeletogenesis (1, 3). In BMPR-I and BMPR-II mutant mice, embryos are unable to develop and lack a mesoderm, indicating BMP signaling is necessary for development of the mesoderm layer (4, 5). BMPs have been shown to also regulate hematopoietic stem cells (HSCs) in the bone marrow and control the size of the HSC compartment (6, 7). BMPs regulate myeloid potential indirectly through stromal osteoblast lineages for increased homing of HSCs in bone marrow (8, 9). Acute lymphoblastic leukemia cells produce BMP-4 to impair differentiation of macrophages and dendritic cells, and maintain a unique pro-tumorigenic microenvironment (10). BMP-2 ligand promotes immunomodulation of macrophages and their induction of bone marrow stroma ontogenesis (11). The role of BMPs in bone formation and hematopoiesis has been well-studied, yet during cancer progression the function of BMPs is an emerging field.

BMPs have divergent roles in cancer, acting as both suppressors and promoters of tumor progression under different circumstances. Based on the cell type and surrounding tumor microenvironment, BMPs take on differing actions in tumor biology (12). A positive correlation exists between BMP expression and clinical stages of cancer in human patients (13). BMPs promote tumorigenesis and progression by driving tumor invasion and angiogenesis, as well as supporting a pro-tumorigenic microenvironment and metastasis (14). Our previous work identified BMPs as a viable target in the tumor and microenvironment, with the BMP inhibitor dorsomorphin homolog 1 (DMH1) reducing tumor progression and metastasis in a breast cancer mouse model (15). Conditional knockout of BMPR1a in a mammary tumor mouse model delayed tumor initiation and prolonged survival (16). Inhibition of BMP signaling impedes M2 polarization of macrophages, supporting an anti-tumorigenic breast cancer microenvironment (15). Our goal was to investigate the impact of BMP signaling inhibition in myeloid cells in a prostate cancer mouse model. Under precise conditions, BMPs exhibit a tumor promoting role in prostate cancer, driving proliferation and invasion (17). BMP signaling in prostate cancer drives bone metastasis, which is the most common site of metastases for prostate cancer patients (18). The LNCaP human prostate cancer cell line exhibits increased proliferation upon BMP-2 treatment in the absence of androgen, however when treated with androgen, BMP-2 inhibited cell growth (19). Apoptosis is induced by BMP signaling in several cancer cell types, but can also be dependent on the surrounding microenvironment to inhibit tumor growth (20). In the PC-3 and DU-145 human prostate cancer cell lines, BMP-7 induces *p21*^*CIP*1/*WAF*1^ to inhibit proliferation and tumor growth (21). BMP-6 has also been found to inhibit growth in DU-145 cells by inducing upregulation of *p21*^*CIP*1/*WAF*1^, *p18*, and *p19* (22). In breast cancer, BMPs elicit dual roles, which depend on specific cell types and conditions that require further investigation (18).

In our study, we utilized a LysMCre mediated myeloid specific BMPR1a conditional knockout mouse model along with a syngeneic prostate tumor model. We show that BMPR1a in myeloid cells plays a pro-tumorigenic role in prostate tumor growth, and that loss of BMPR1a impairs tumor progression. Myeloid differentiation in the bone marrow and spleen also exhibit alterations to the immune compartments upon loss of myeloid BMPR1a signaling. Utilizing the pharmacologic BMP inhibitor DMH1, we found a requirement for polarization of M2 macrophages. Our findings suggest that inhibiting BMPR1a signaling may be a viable therapeutic approach for prostate cancer patients.

## Materials and Methods

### Cell Culture

The THP-1 cell line was obtained from the American Type Culture Collection (ATCC) and cultured in RPMI-1640 Medium (Corning) and 10% Fetal Bovine Serum (FBS) (Seradigm). The Raw264.7 cell line was obtained from ATCC and cultured in Dulbecco's Modified Eagle Medium (DMEM) high glucose with sodium pyruvate (Corning) and 10% FBS (Seradigm). The MyC-CaP cell line was obtained from Dr. Austin Kirschner at Vanderbilt University with permission of ATCC and cultured in DMEM (Corning) and 10% FBS (Seradigm). All cell lines were routinely tested for mycoplasma infection by PCR and authenticated by morphology and published growth rates available from ATCC.

### Mouse Models

LysMCre (Jax Stock #004781), BMPR1a floxed (MMRRC UNC STOCK #030469), tdTomato, -EGFP (mTmG) Cre reporter (Jax Stock # 007676) mice were bred onto an FVBn (Jax Stock # 001800) background. Six week old male FVBn mice expressing LysMCre^+^.BMPR1a^*wt*^.mTmG^+^ (Control) or LysMCre^+^.BMPR1a^*fl*/*fl*^.mTmG^+^ (cKO) were used in tumor naïve and tumor bearing experiments (23, 24). All mice contained at least one allele of the tdTomato/EGFP Cre reporter of recombination (25). For tumor naïve studies, the spleen and bone marrow were harvested for experimental analysis. For the tumor studies, 1 × 10^5^ MyC-CaP cells in 100 μL PBS were injected subcutaneously into both flanks for each mouse. Tumors were palpable at 15 days post injection and tumor volume (length × width) was measured with calipers every 2–3 days for 29 days. Once tumors reached the maximum acceptable size at day 29, mice were sacrificed and the spleen, bone marrow, and tumors were harvested for experimental analysis. LysMCre^+^.BMPR1a^*fl*/*wt*^.mTmG^+^ mice were also maintained on a C57Bl6 background for primary cell line development. Mice were bred and maintained at Vanderbilt University and the Nashville Veterans Affairs Medical Center (protocol number V/16/012) as well as at the University of Colorado Anschutz Medical Campus (protocol number 00553). All animal procedures were performed in accordance with the National Institutes of Health's Guide for the Care and Use of Laboratory Animals and were approved by the Institutional Animal Care and Use Committees.

### Primary Bone Marrow Derived BMPR1a Control and cKO Cell Lines

The BMPR1a cell lines were derived from 5 month old male C57Bl6 mice with the following genotypes:

LysMCre^+^.BMPR1a^wt/wt^.mTmG^+/−^ (Control) will be referred to as PODS4 and LysMCre^+^.BMPR1a^flox/flox^ .mTmG^+/−^ (cKO) will be referred to as PODS5. Femur and tibia bones were harvested from mice, sliced lengthwise and placed in a T-75 vented tissue culture treated flask (Greiner) with 20 mL of DMEM (Corning) supplemented with 10% FBS (Seradigm) and 3X antibiotic (Gibco). Cells were cultured in a humidified incubator at 37°C and 5% CO_2_. Media was changed at 48 h, then the bone fragments were removed between day 7 and 10 of culture. Cells were expanded into two flasks prior to flow cytometry sorting. Sorting was performed by the CU Cancer Center Flow Cytometry Shared Resource using a MoFlo XDP Cell Sorter (Beckman Coulter) with a 100 μm nozzle tip. After multiple flow cytometry sorts, PODS4 double positive cells expressing tdTomato and EGFP were collected, and PODS5 single positive cells expressing EGFP were collected. Both cell lines were expanded for macrophage polarization experiments. Cell morphology and EGFP expression was assessed at 20X on an Eclipse Ni inverted microscope (Nikon) (Supplemental Figure 3B).

### Histology and Immunohistochemistry (IHC)

*Ex vivo* tissues were harvested and immediately placed in 10% formalin and fixed for 24 h. Tumors were butterflied and then laid flat in a cassette to ensure the center of the tumors were reached when sectioning. Then formalin was replaced with 70% ethanol for 24 h prior to embedding in paraffin wax. Formalin-fixed and paraffin-embedded (FFPE) tissue blocks were sectioned at 5 μm thickness with one section per stain, and mounted on plus coated microscope slides. Unstained slides were baked for 1 h at 60° Celsius prior to paraffin removal with xylenes and rehydration of tissue in graduated ethanol rinses (100-95-70-50-PBS). Antigen retrieval was performed in Citrate pH 6.0 for heat-induced epitope retrieval. Routine H&E staining was performed in Harris hematoxylin (Vector Labs). Primary antibodies for F4/80 (Bio-Rad 1:200), Ki-67 (Sigma 1:500), Cleaved Caspase-3 (Cell Signaling 1:100), BMPR1a (Millipore 1:100) and pSMAD1/5/8 (Millipore 1:100) were used for immunohistochemistry (IHC) staining. Signal was detected by ImmPRESS polymer secondaries to appropriate host and DAB chromogen substrate (Vector Labs) and counterstained with Hematoxylin QS (Vector Labs). All bright field IHC and H&E were scanned at 40X (0.22 um/pixel) magnification using a ScanScope XT System (Aperio Technologies). To quantitate IHC staining, a grid of up to 5 20X images per tumor were captured, avoiding excessive stroma or necrotic tissues, on an Eclipse Ni microscope (Nikon) and imported into ImageJ (U.S. National Institutes of Health) to change contrast to blue and auto-adjust threshold then measure the mean staining. The mean measurement was then averaged across all images for each tumor.

### Flow Cytometry

Single cell suspension of spleens were prepared by crushing the spleen between two microscope slides and filtered in a 70 μm cell strainer. Single suspension of bone marrow was prepared by removing femurs from mice and flushing PBS from a 25 gauge tuberculin syringe through the marrow cavity and filtered by a 70 μm cell strainer. Single cell suspension of tumor were digested in neutral protease (Worthington Bio), collagenase 3 (Worthington Bio), DNase (Worthington Bio) and 3X antibiotic (ThermoFisher Scientific). Prior to staining, all cells were frozen in 90% FBS (Seradigm) and 10% dimethyl sulfoxide (DMSO) (MP Biomedicals) and stored at −80°C to allow for more uniform staining of samples and immediate analysis without time limitations. Frozen single cells were thawed in a 37°C water bath for 3 min then washed in FACS buffer before staining. The single cells were blocked in 5% FBS (Seradigm), then stained for surface markers and filtered prior to acquisition on the Fortessa cytometer (BD Biosciences). The antibodies used are listed in Supplemental Table 2 (Biolegend). Flow cytometry data analysis was performed on FlowJo (version 10 for Windows, BD Biosciences). The gating strategy is shown in Supplemental Figure 1A.

### CyTOF

Single cell suspension of spleens were prepared by crushing the spleen between two microscope slides then washing with PBS and filtering in a 70 μm cell strainer. Single suspension of bone marrow was prepared by removing femurs from mice and flushing PBS from a 25 gauge tuberculin syringe through the marrow cavity and filtered by a 70 μm cell strainer. Single cell suspension of tumor were digested in neutral protease (Worthington Bio), collagenase 3 (Worthington Bio), DNase (Worthington Bio), and 3X antibiotic (ThermoFisher Scientific). Prior to staining, all cells were frozen in 90% FBS (Seradigm) and 10% DMSO (MP Biomedicals) and stored at −80°C to allow for more uniform staining of samples and immediate analysis without time limitations. Frozen single cells were thawed in a 37°C water bath for 3 min then washed in PBS before staining. Single cells were stained for surface markers then intracellular markers and filtered prior to acquisition on the Mass Cytometer (Fluidigm). Staining and acquisition was performed by the Vanderbilt University Cancer and Immunology Core. The antibodies used are listed in Supplemental Table 3 (Fluidigm). High dimensional CyTOF analysis was performed on Cytobank (premium.cytobank.org) and viSNE analysis was performed with implementation of 1000 iterations, a perplexity of 30, and a theta of 0.5. For viSNE clustering individual sample FCS files were concatenated based on tissue, BMPR1a genotype, and tumor or naïve, then analysis was run for all stained channels. The gating strategy is shown in Supplemental Figure 1B. Cells were gated off of a DNA+ gate, followed by a Live Cell gate then a CD45^+^ gate to isolate immune cells. viSNE clustering was implemented on all CD45^+^ cells and included all staining markers, with gates manually drawn around immune cell populations based on positive staining for each immune marker from the viSNE clusters.

### Macrophage Polarization With BMP Inhibition

THP-1 cells were plated at 2 × 10^5^ cells in triplicate 6-well cell culture plates (Greiner Bio-One) in 2 mL of culture media and incubated for 48 h with 1 ug/mL phorbol 12-myristate 13-acetate (PMA) (Millipore) to activate suspension monocytes into differentiated adherent macrophages, along with 100 ng/mL human IL-4 (RnD), and 100 ng/mL human IL-13 (RnD) to polarize cells into M2 macrophages. Adherent Raw264.7 cells were plated in triplicate at 1 × 10^5^ cells in 6-well cell culture plates (Greiner Bio-One) in 2 mL culture media and incubated for 48 h with 100 ng/mL mouse IL-4 (Biolegend), and 100 ng/mL mouse IL-13 (Biolegend) to polarize the macrophages into M2 macrophages. Both THP-1 and Raw264.7 cells were simultaneously treated with 10 uM DMH1 (Selleckchem) (26) or DMSO (MP Biomedicals) control. PODS4 and PODS5 cells were plated in triplicate at 2.5 × 10^5^ cells in 2 mL culture media per well of 6-well cell culture plates (Greiner Bio-One) and incubated for 48 h with 100 ng/mL mouse IL-4 (Biolegend), and 100 ng/mL mouse IL-13 (Biolegend) to polarize the macrophages into M2 macrophages. No PMA was used to activate adherent Raw264.7, PODS4, and PODS5 cell lines into M2 macrophages. After 48 h of incubation, cells were lysed and RNA was purified using the RNeasy Plus Mini Kit (Qiagen). RNA quality was assessed using Nanodrop 2000 Spectrometer (ThermoFisher Scientific) and samples with a 260/280 ratio above 1.6 were used.

### Gene Expression

The iScript cDNA Synthesis Kit (Bio-Rad) was used to generate cDNA from 1 μg of total RNA. Real-time PCR reactions were performed using the SsoAdvanced Universal SYBR Green Supermix (Bio-Rad) on a CFX QPCR instrument (Bio-Rad). The targets and primers used are listed in Supplemental Table 1. All genes were run in technical and biological triplicate, with glyceraldehyde 3-phosphate dehydrogenase (*GAPDH*) as the housekeeping gene to normalize gene expression.

### Statistics

Statistical analyses were performed using GraphPad Prism (version 7.04 for Windows; GraphPad Software Inc.) and Excel (version 2016 for Windows; Microsoft Corp.). All statistical tests used a cutoff *p*-value of 0.05 for significance and were two-sample one-tailed student *t*-tests with assumed heteroscedasticity. One-tailed tests were used to compare values that are all > 0 and to enhance the power to reject the null hypothesis if the null hypothesis is false (27).

## Results

### Loss of Myeloid Cell BMPR1a Alters the Myeloid Compartment

**Figure 1:**
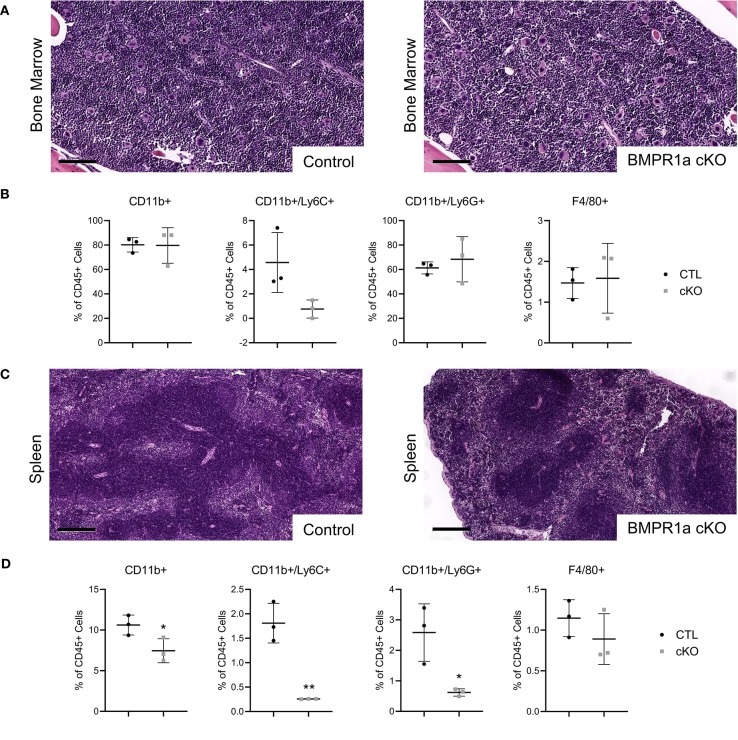
Loss of myeloid cell BMPR1a alters the bone marrow and spleen compartment. Bone marrow and spleens from tumor naïve control (CTL) and LysMCre BMPR1a knockout (cKO) male mice were harvested. **(A)** H&E staining of bone marrow from naïve CTL and cKO mice. Scale bars indicate 100 μm. **(B)** Flow cytometry analysis of bone marrow from naïve CTL and cKO mice. Viability for naïve bone marrow ranged from 7.33 to 14.8%. Mean graphed with SD, ^*^indicates statistical significance *p* ≤ 0.05 and ^**^indicates *p* ≤ 0.01 by Student *t*-test. **(C)** H&E staining of spleen from naïve CTL and cKO mice. Scale bars indicate 200 μm. **(D)** Flow cytometry analysis of spleen from naïve CTL and cKO mice. Viability for naïve spleen ranged from 6.93 to 37.7%. Mean graphed with SD, ^*^indicates statistical significance *p* ≤ 0.05 and ^**^indicates *p* ≤ 0.01 by Student *t*-test.

To investigate the role of myeloid BMPR1a in mouse models, control (CTL) and conditional BMPR1a knockout (cKO) mice were generated in the FVBn background. Under the myeloid specific LysMCre promoter, BMPR1a was deleted in cKO mice, or a control lacking floxed alleles was used for CTL mice. To observe differences in the myeloid compartment dependent on BMPR1a loss, bone marrow and spleens were harvested from 6 week old male mice. Histological analysis of bone marrow from CTL and BMPR1a cKO mice displayed similar morphologies (Figure 1A). Flow cytometry analysis of tissues highlighted changes in the myeloid populations in the bone marrow (Figure 1B). In the bone marrow of BMPR1a cKO mice, CD11b+/Ly6C+ monocytes were reduced (Figure 1B). Histological analysis of spleen from CTL and BMPR1a cKO mice displayed similar morphologies (Figure 1C). Flow cytometry analysis of spleens highlighted changes in the myeloid populations (Figure 1D). In the spleen, clear changes where observed upon BMPR1a knockout in myeloid cells, with significant reduction for CD11b+ myeloid cells, CD11b+/Ly6C+ monocytes, and CD11b+/Ly6G neutrophils (Figure 1D).

### Loss of Myeloid Cell BMPR1a Produces Unique Innate Immune Clusters

To further explore the alternations in myeloid populations after conditional BMPR1a knockout, we used mass cytometry (CyTOF) to identify discrete changes in myeloid populations upon loss of BMPR1a. CyTOF analysis of samples allowed expanded assessment of phenotypic and functional changes of single immune cells compared to flow cytometry, with enhanced clustering of high-dimensional analysis (28). CyTOF analysis with viSNE clustering rather than biaxial gating in flow cytometry allows for smaller cellular associations to be identified and reduces the risk of gating out rare cell populations (29). Bone marrow and spleen were collected from 6 week old male CTL and BMPR1a cKO mice. CyTOF staining with a panel of 26 immune markers (Supplemental Table 3) produced mixed staining for intracellular cytokines, with more robust staining for surface immune phenotyping markers. To generate viSNE clusters, analysis was performed on Cytobank where cells were gated off of a DNA+ gate, followed by a live cell gate then a CD45+ gate to isolate immune cells (Supplemental Figure 1B). viSNE clustering was implemented on all CD45+ cells and included all staining markers, with gates for CD19+/CD20+ B cells, CD4+ T cells, CD8+ T cells, CD69+ T cells, Gr1+ (Ly6GC+) cells, CD11b+ cells, and CD11c+ dendritic cells. Despite an increase in parameters from flow cytometry to CyTOF analysis, commercial antibodies are still limited for CyTOF staining, resulting in the Ly6C and Ly6G expression analyzed by flow cytometry being replaced with Gr1 for CyTOF. The resulting viSNE depicts overlaying clusters based on co-expression of immune markers.

**Figure 2:**
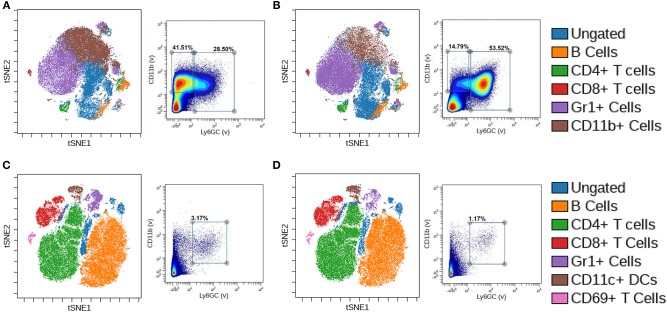
Loss of myeloid cell BMPR1a produces unique innate immune clusters. Bone marrow and spleens from three control (CTL) and three LysMCre BMPR1a knockout (cKO) male mice were harvested. Cells were stained with a 26 antibody panel for mouse immune cell identification and cytokine signaling and run on the Helios CyTOF. viSNE analysis was preformed to identify unique immune cell clusters. **(A)** viSNE of naïve bone marrow from CTL mice (left), and biaxial gate (right) from viSNE of Gr1 and CD11b staining. Viability for CTL naïve bone marrow was 99.11%. **(B)** viSNE of naïve bone marrow from cKO mice (left), and biaxial gate (right) from viSNE of Gr1 and CD11b staining. Viability for cKO naïve bone marrow was 98.75%. **(C)** viSNE of naïve spleen from CTL mice (left), and biaxial gate (right) from viSNE of Gr1 and CD11b staining. Viability for CTL naïve spleen was 95.6%. **(D)** viSNE of naïve spleen from cKO mice (left), and biaxial gate (right) from viSNE of Gr1 and CD11b staining. Viability for cKO naïve spleen was 95.69%.

In the naïve bone marrow of CTL mice, B cells, CD4+ and CD8+ T cells, Gr1+ cells, and CD11b+ cell clusters were identified (Figure 2A). Based on biaxial gating from naïve CTL bone marrow on Gr1 and CD11b, two populations emerged—a Gr1–/CD11b+ gate of 41.51% of cells, and a Gr1+/CD11b+ gate containing 28.50% of cells. We observed alterations to the differentiation and lineage capability of immune cells in the bone marrow upon myeloid BMR1a loss (Figure 2B). The CD11b+ cell cluster decreased while the Gr1+ cluster increased in cKO mouse bone marrow. Biaxial gating on Gr1 and CD11b staining inversed, with lower Gr1–/CD11b+ cells at 14.79% and increased in Gr1+/CD11b+ cells to 53.52% in the cKO bone marrow. In the naïve spleen of CTL mice, B cells, CD4+, CD8+, and CD69+ T cells, along with Gr1+ cells and CD11c+ cell clusters were characterized (Figure 2C). A double positive Gr1+/CD11b+ cell population was identified in the spleen, with 3.17% of cells from naïve CTL spleens. Knocked out BMPR1a in myeloid cells altered clustering of immune cells in cKO spleens (Figure 2D). The Gr1+ cell cluster was decreased, along with the biaxial gating on Gr1+/CD11b+ cells, reducing the population to 1.17% of total cells.

### Prostate Tumor Growth Is Restricted in Myeloid BMPR1a Knockout Mice

**Figure 3:**
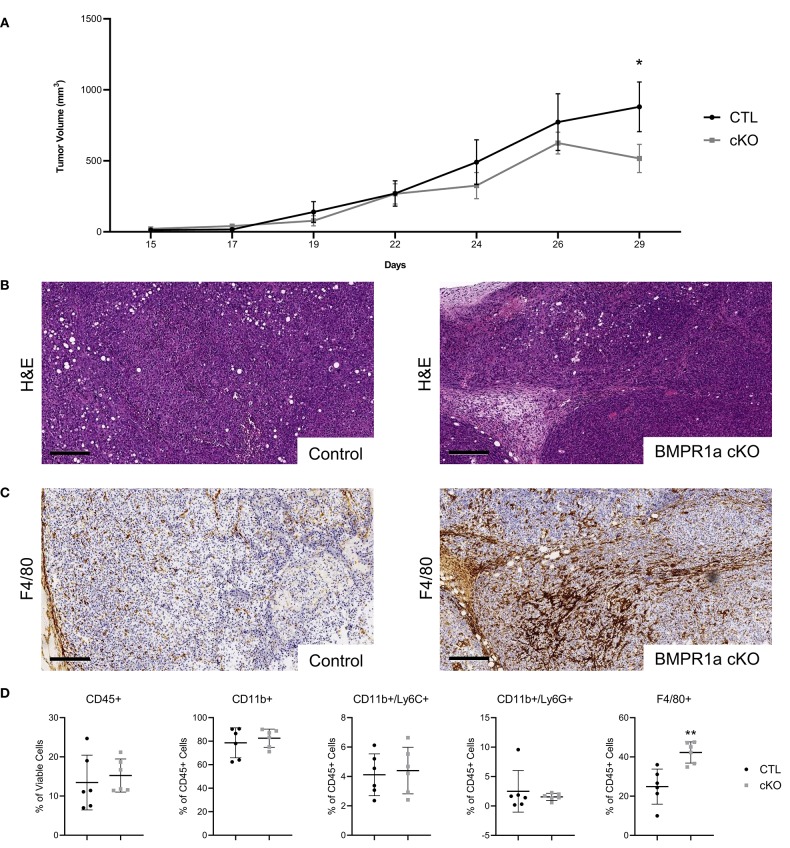
Prostate tumor growth is restricted in myeloid BMPR1a knockout mice. 1 × 10^5^ MyC-CaP cells were injected into both flanks of six control (CTL) syngeneic mice and six LysMCre BMPR1a knockout (cKO) syngeneic mice (*n* = 12). Tumors were palpable at 15 days post injection then were allowed to grow for two additional weeks before tumors reached maximum acceptable size. **(A)** Flank tumor volume decreases in cKO mice. Tumor volume determined by caliper measurements for height, width and length. Mean graphed with SEM, ^*^indicates statistical significance *p* ≤ 0.05 by Student *t*-test. **(B)** H&E staining of tumors. Scale bars indicate 200 μm. **(C)** IHC staining of tumors for F4/80 at day 29. Scale bars indicate 200 μm. **(D)** Flow cytometry analysis of tumor from CTL and cKO mice. Viability for tumor ranged from 34.4 to 67.8%. Mean graphed with SD, ^*^indicates statistical significance *p* ≤ 0.05 and ^**^indicates *p* ≤ 0.01 by Student *t*-test.

We next wanted to examine if BMPR1a loss in myeloid cells influences prostate cancer progression. The mouse FVBn syngeneic prostate cancer cell line MyC-CaP was subcutaneously injected into the flank of CTL and BMPR1a cKO male mice. The MyC-CaP cell line is unique because it is an androgen dependent model of mouse prostate adenocarcinoma, while the majority of available prostate cancer lines are from human patients and androgen independent due to hormone treatment (30). Orthotopic and subcutaneous prostate cancer mouse models using MyC-CaP cells have been extremely informative in expanding syngeneic tumor studies (31, 32). After 15 days, the tumors were palpable and growth was monitored by calipers. Tumor volume progressed until tumors reached the maximum acceptable size (2 cm in any direction) at 29 days (Figure 3A). The growth of these MyC-CaP tumors correlates with previous tumor studies, with an increase in tumor volume without significant alterations to tumor proliferation until day 29 (32). At day 29, the BMPR1a cKO tumor volume reduction was statistically significant (*p* = 0.05) (Figure 3A). At the endpoint of study, the tumors were resected and analyzed by histology for H&E (Figure 3B). Analysis of tumor pathology revealed subtle changes in morphology of the tumors from the CTL mice compared to the BMPR1A cKO (Figure 3B). Staining for macrophages expressing F4/80 by IHC showed BMPR1a cKO tumors exhibited strong macrophage infiltration (Figure 3C). Flow cytometry analysis of the tumors confirmed significant increase (*p* = 0.05) in macrophage F4/80+ staining in the BMPR1a cKO tumor mice compared to control (Figure 3D). To assess if BMPR1a myeloid loss altered proliferation and cell death in the tumor, tumor sections were stained for Ki-67 and cleaved caspase-3 by IHC. No change in proliferation was observed in CTL and BMPR1a cKO by Ki-67 staining (*p* = 0.94) (Supplemental Figures 2A,B). Staining for apoptosis with cleaved caspase 3 did not change between CTL and BMPR1a cKO tumors (*p* = 0.17) (Supplemental Figures 2C,D). Tumors were also stained for BMPR1a and pSMAD1/5/8 by IHC to determine if myeloid BMPR1a deletion impacts BMPR1a expression and signaling in the tumor microenvironment. Staining for BMPR1a and pSMAD1/5/8 was heterogeneous within the tumor and surrounding stroma, with no difference in staining density between CTL and cKO tumors (Supplemental Figures 2E,D).

### Loss of Myeloid Cell BMPR1a Alters the Myeloid Compartment in Tumor Bearing Mice

**Figure 4:**
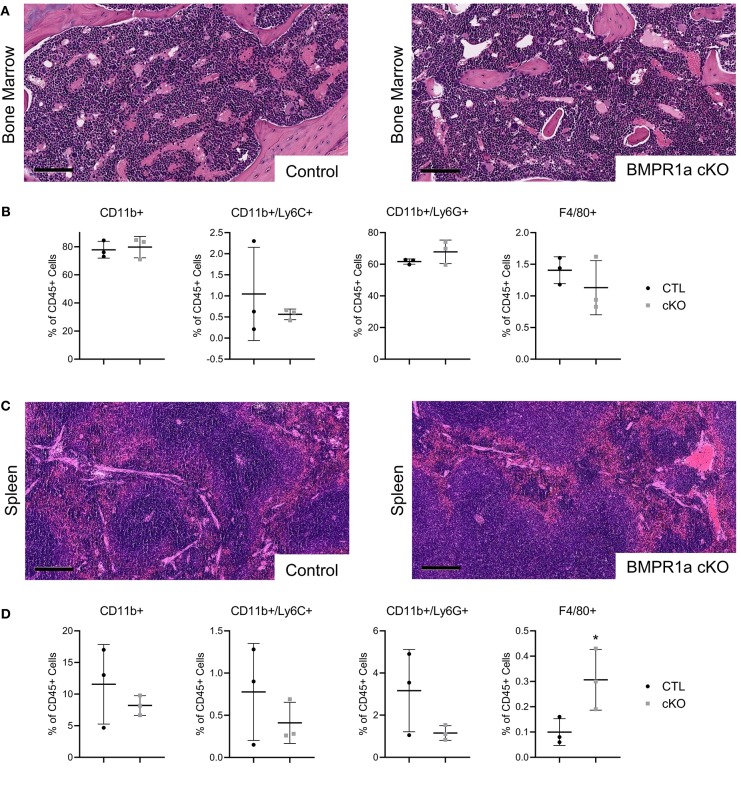
Loss of myeloid cell BMPR1a Alters the bone marrow and spleen compartment in tumor bearing mice. Bone marrow and spleens from MyC-CaP flank tumor bearing control (CTL) and LysMCre BMPR1a knockout (cKO) male mice were harvested. **(A)** H&E staining of bone marrow from tumor bearing CTL and cKO mice. Scale bars indicate 100 μm. **(B)** Flow cytometry analysis of bone marrow from tumor bearing CTL and cKO mice. Viability for tumor bearing bone marrow ranged from 21.5 to 30.6%. Mean graphed with SD, ^*^indicates statistical significance *p* ≤ 0.05 and ^**^indicates *p* ≤ 0.01 by Student *t*-test. **(C)** H&E staining of spleen from tumor bearing CTL and cKO mice. Scale bars indicate 200 μm. **(D)** Flow cytometry analysis of spleen from tumor bearing CTL and cKO mice. Viability for tumor bearing spleen ranged from 6.93 to 29%. Mean graphed with SD, ^*^indicates statistical significance *p* ≤ 0.05 by student *t*-test.

We evaluated structure and composition changes in bone marrow and spleen to determine if BMPR1a loss alters the myeloid compartments of tumor bearing mice. Histology of the bone marrow from the tumor mice appeared to have the same structure and pathology in both control and knockout (Figure 4A). Using flow analysis for immune cell markers from the bone marrow, the populations of CD11b+ myeloid, CD11b+/Ly6C+ monocyte, CD11b+/Ly6G+ neutrophil, and F4/80+ macrophages were unchanged between CTL and cKO tumor mouse groups (Figure 4B). In the spleen, histological analysis showed no change in structure and pathology between control and knockout tumor mice (Figure 4C). Flow analysis for immune cells in the spleen of tumor bearing mice exhibited no change in CD11b+ myeloid, CD11b+/Ly6C+ monocyte and CD11b+/Ly6G+ neutrophil populations, but showed a significant increase in F4/80+ macrophages in the BMPR1a cKO tumor mice (Figure 4D).

### Loss of Myeloid Cell BMPR1a Produces Unique Innate and Adaptive Immune Clusters in Tumor Bearing Mice

**Figure 5:**
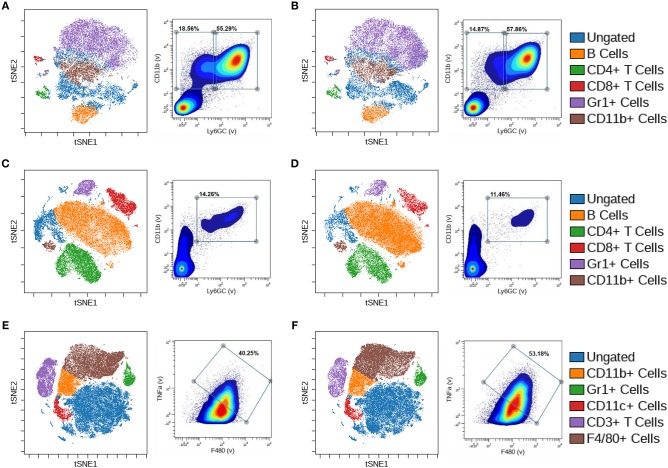
Loss of myeloid cell BMPR1a produces unique innate and adaptive immune clusters in tumor bearing mice. Bone marrow, spleens, and tumors from control (CTL) and LysMCre BMPR1a knockout (cKO) male mice with flank MyC-CaP tumors were harvested. Cells were stained with a 26 antibody panel for mouse immune cell identification and cytokine signaling and run on the Helios CyTOF. viSNE analysis was preformed to identify unique immune cell clusters. **(A)** viSNE of bone marrow from tumor bearing CTL mice (left), and biaxial gate (right) from viSNE of Gr1 and CD11b. Viability for CTL tumor bearing bone marrow was 83.97%. **(B)** viSNE of bone marrow from tumor bearing cKO mice (left), and biaxial gate (right) from viSNE of Gr1 and CD11b staining. Viability for cKO tumor bearing bone marrow was 82.7%. **(C)** viSNE of spleen from tumor bearing CTL mice (left), and biaxial gate (right) from viSNE of Gr1 and CD11b staining. Viability for CTL tumor bearing spleen was 53.15%. **(D)** viSNE of spleen from tumor bearing cKO mice (left), and biaxial gate (right) from viSNE of Gr1 and CD11b staining. Viability for cKO tumor bearing spleen was 64.59%. **(E)** viSNE of tumor from tumor bearing CTL mice (left), and biaxial gate (right) from viSNE of F4/80 and TNFα staining. Viability for CTL tumor was 41.21%. **(F)** viSNE of tumor from tumor bearing cKO mice (left), and biaxial gate (right) from viSNE of F4/80 and TNFα staining. Viability for cKO tumor was 40.09%.

CyTOF analysis of the tumor, bone marrow, and spleen of control and BMPR1a knockout mice was performed to identify changes in immune cell population clusters. The same 26 immune marker panel and gating strategy was performed again to generate viSNE clusters (Supplemental Table 3 and Supplemental Figure 1B). viSNE clustering was implemented on all CD45+ cells, with gates for CD19+/CD20+ B cells, CD4+ T cells, CD8+ T cells, CD69+ T cells, CD3+ T cells, Gr1+ cells, CD11b+ cells, CD11c+ dendritic cells, and F4/80+ macrophages. The bone marrow of tumor bearing mice exhibited B cell, CD4+ T cell, Cd8+ T cell, Gr1+ cell, and CD11b+ cell clusters (Figures 5A,B). Biaxial gating on Gr1 and CD11b expression for bone marrow from CTL tumor mice exhibited two distinct populations, 18.56% Gr1-/CD11b+ and 55.29% Gr1+/CD11b+ (Figure 5A). In cKO tumor bearing bone marrow, no distinct changes in viSNE clusters were observed (Figure 5B). However, the Gr1-/CD11b+ biaxial gated cell proportion was decreased to 14.87% in the bone marrow from cKO tumor bearing mice. In the spleen, the same immune cell lineages were present as in the bone marrow but clustering alterations were more pronounced (Figures 5C,D). One immune cell population was identified that was 14.26% Gr1+/CD11b+ in the CTL spleen (Figure 5C). In BMPR1a cKO tumor bearing spleens, Gr1+ cell, CD4+ T cell, and CD8+ T cell clustering was decreased while the B cell cluster increased (Figure 5D). The cKO spleen Gr1+/CD11b+ population decreased to 11.46% of cells. The MyC-CaP tumors displayed unique clusters for CD11b+ cells, Gr1+ cells, CD11c+ cells, CD3+ T cells, and F4/80+ cells (Figures 5E,F). A unique population of 40.25% F4/80+/TNFα+ M1-like macrophages was found in CTL mouse tumors (Figure 5E). In the tumors of BMPR1a cKO mice, no significant alterations to clustering was observed, but an increase to 53.18% F4/80+/TNFα+ M1-like macrophages indicated a shift of macrophages toward M1 polarization (Figure 5F).

### Macrophage Polarization Is Altered by BMPR1a Inhibition

**Figure 6:**
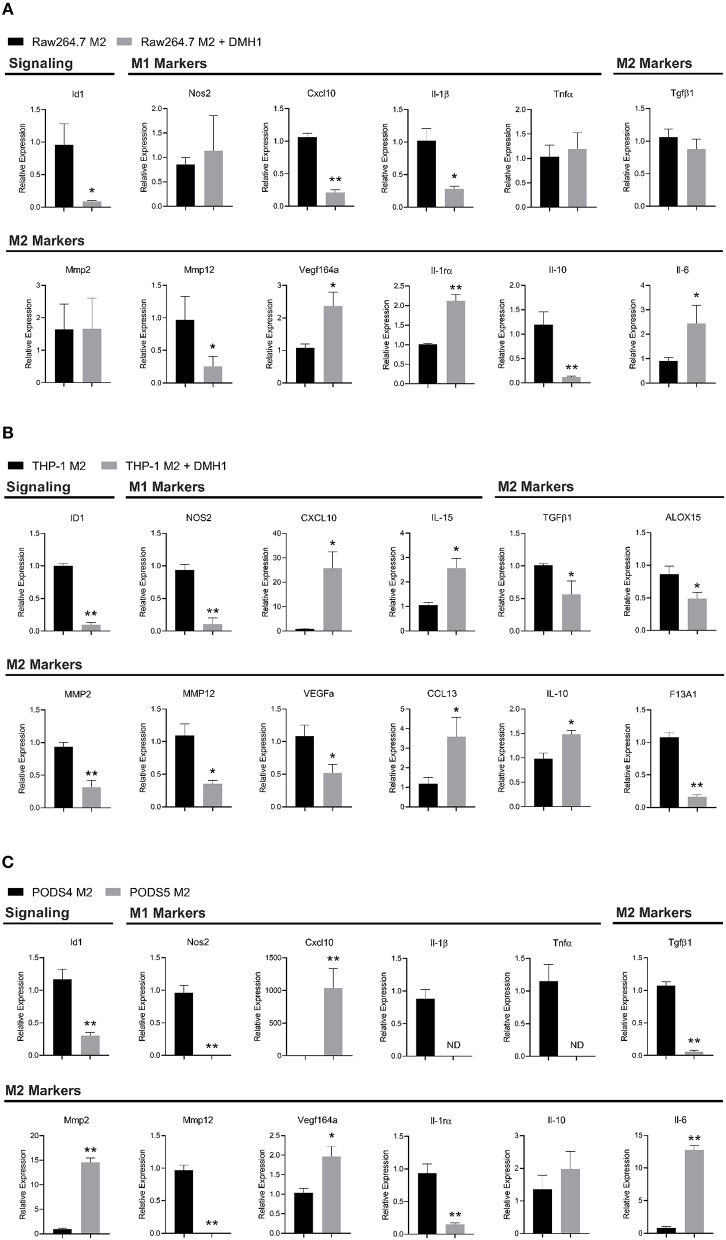
Macrophage polarization is altered by BMPR1a inhibition. Myeloid cell lines were polarized into M2 macrophages and treated with the BMPR1a inhibitor DMH1 for 48 h. RNA was isolated and RT-PCR was run to assess gene expression of BMPR1a target genes and macrophage polarization markers. **(A)** Gene expression of BMP signaling and M1/M2 polarization for Raw264.7 cell line polarized into M2 macrophages with DMH1 or DMSO treatment in triplicate. Mean graphed with SD, ^*^indicates statistical significance *p* ≤ 0.05 and ^**^indicates *p* ≤ 0.01 by student *t*-test. **(B)** Gene expression of BMP signaling and M1/M2 polarization for THP-1 cell line polarized into M2 macrophages with DMH1 or DMSO treatment in triplicate. Mean graphed with SD, ^*^indicates statistical significance *p* ≤ 0.05 and ^**^indicates *p* ≤ 0.01 by student *t*-test. **(C)** Gene expression of BMP signaling and M1/M2 polarization for PODS4 and PODS5 cell lines polarized into M2 macrophages in triplicate. Mean graphed with SD, ND indicates sample not detected and is below the limits of detection, ^*^indicates statistical significance *p* ≤ 0.05 and ^**^indicates *p* ≤ 0.01 by student *t*-test.

To further investigate BMP dependent macrophage polarization, macrophage cell lines were polarized into a M2 phenotype and treated with BMP inhibitor DMH1. DMH1 was selected due to its higher specificity for BMP type I receptors compared to other BMP inhibitors including Dorsomorphin and its analog LDN-193189 (33). In mouse Raw264.7 M2 macrophages, DMH1 treatment resulted in distinct changes in polarization markers by RT-PCR. A panel of both M1 (*Il-1*β, *Tnf*α, *Cxcl10, Nos2*) and M2 (*Il-10, Tgf*β*1, IL-1r*α, *Vegf164a, Il-6, Mmp2*, and *Mmp12*) canonical and emerging polarization markers were used to highlight the distinct molecular phenotypes of BMP signaling inhibition in mouse cells (34). With the inhibition of BMP, we were able to see a reduction in *Id1*, a downstream effector of BMP signaling in Raw264.7 cells (Figure 6A). DMH1 treatment resulted in a variety of changes in mouse Raw264.7 M2 macrophages, increasing expression of some M2 markers (*Vegf164a, Il-1r*α, *Il-6*), while decreasing in others (*Il-10, MMP-12*) (Figure 6A). Mouse Raw264.7 M2 macrophages also showed a decrease in *Cxcl10* and *Il-1*β, both M1 macrophage markers, after inhibition of BMP signaling.

In the human THP-1 monocyte cell line, M2 activation with DMH1 treatment showed distinct results (Figure 6B). Human macrophage polarization markers assessing M1 (*CXCL10, IL-15* and *NOS2*) and M2 (*IL-10, CCL13, TGF*β*1, MMP2, MMP12, ALOX15, VEGFa*, and *F13A1*) canonical and emerging biomarkers were used to identify alterations upon BMP inhibition (35). BMP inhibition in M2 polarized THP-1 cells resulted in several M2 markers increasing expression (*IL-10* and *CCL13*), while others decreased (*TGF*β*1, MMP2, MMP12, ALOX15, VEGFa*, and *F13A1*). M1 markers were equally divergent. *CXCL10* and *IL-15* expression increased while *NOS2* expression decreased upon DMH1 treatment.

To reconcile the results of our mouse Raw264.7 and human THP-1 cell line M2 polarization, we turned to our genetic BMPR1a deletion mouse model. We generated primary bone marrow derived cell lines from control and cKO tumor naïve mice from the C57Bl6 background (Supplemental Figure 3A). These cell lines, referred to as PODS4 for the control myeloid cells and PODS5 for the cKO myeloid cells, both exhibited similar morphologies and expressed EGFP indicating recombination of the LysMCre (Supplemental Figure 3B). Both cell lines were sorted by flow cytometry to collect the double positive tdTomato and EGFP population for PODS4 while PODS5 were sorted to collect the single positive EGFP population (Supplemental Figure 3C). PODS4 and PODS5 were then polarized into a M2 phenotype and assessed for BMP signaling and polarization changes. The BMP effector *Id1* was significantly decreased in PODS5 M2 activated cells (Figure 6C). Interestingly, the other BMP receptors *Acvr1* (*Alk-2*), *Bmpr1b* (*Alk-6*), and *Bmpr2* were not universally altered upon BMPR1a deletion. No change in *Acvr1* and *Bmpr1b* expression was observed, although the relative abundance of *Bmpr1b* was at the lower limits of detection (Supplemental Figure 3D). *Bmpr2* was reduced in the PODS5 M2 cells by 2.3 fold (Supplemental Figure 3D). BMPR1a knockout in PODS5 decreased the expression of M1 markers *Nos2, Il-1*β and *Tnf*α, while *Cxcl10* was highly upregulated (Figure 6C). M2 markers exhibited discordant changes in the PODS5, with *Tgf*β*1, MMP12*, and *Il-1r*α decreasing expression while *Il-6, Mmp2*, and *Vegf164a* increased.

Markers of polarization are used to assess if macrophages possess a tumor promoting or tumor suppressing phenotype. Across Raw264.7, THP-1, and PODS polarization markers, only the M2 marker *MMP12* decreased in M2 cells with inhibited BMP signaling in all three cell lines. The remaining polarization genes were not consistent in their expression for the cell lines, with *NOS2* (M1) and *TGF*β*1* (M2) decreasing in THP-1 and PODS cells upon BMPR1a activity inhibition while *Vegf164a* (M2) and *Il-6* (M2) increasing in Raw264.7 and PODS cells. Other markers were unique to each cell line, with *MMP2* (M2) and *IL-10* (M2) exhibiting increased, decreased or no change in expression with BMP inhibition. These findings highlight the complexity of macrophage polarization and signaling, paralleling the context-dependent role of BMPs. This disparate polarization *in vitro* will lead to a greater diversity of a macrophage response *in vivo* given that macrophages in patient tumors are not positionally uniform or solely dependent upon IL-4 and IL-13 stimulation.

## Discussion

The dynamic role of BMPs in cancer have been demonstrated by highlighting the importance of cellular and environmental context when studying BMPs. Lineage commitment of cells are driven by BMPs during development and cancer progression (36). We demonstrate that BMPs alter the composition of myeloid cells in lymphatic organs and modify gene expression polarization markers in M2 polarized myeloid cell lines. In the microenvironment of our primary prostate tumors, BMPR1a signaling status also influenced myeloid populations, exhibiting strong M1 macrophage infiltration in the BMPR1a cKO tumors. Furthermore, pharmacologic inhibition of polarized human and mouse macrophages modulates M2-like gene expression phenotypes.

LysMCre is a useful conditional Cre system, with deletion in macrophages, mature macrophages and neutrophils, along with monocytes and specific inflammatory and resident monocytes populations without significantly affecting other myeloid or lymphoid populations (37). A double-fluorescent Cre reporter was used to ensure Cre activity, with mice expressing membrane tdTomato without recombination, but switch to expressing membrane EGFP upon Cre mediated excision (25). Conditional knockout of BMPR1a was first used two decades after showing that global knockout of BMPR1a results in embryonic lethality (24). Unique phenotypes have been reported in conditional BMPR1a deletion models. Our previous work in mammary gland BMPR1a knockout studies resulted in a unique shift to alternate focal morphologies in the knockout tumors, exhibiting more desmoplastic, carcinoma-like or squamous cell carcinoma-like features (16). While in osteoblasts, bone structure and strength is improved upon BMPR1a deletion (38). Combining all three systems for a LysMCre BMPR1a knockout model with a double-fluorescence reporter enabled our study of BMP signaling in myeloid cells. BMPs are required during hematopoietic precursor development and subsequent lineage expansion (39). In this study, mice lacking BMPR1a via LysMCre deletion were healthy and did not display any gross defects. This was somewhat surprising due to the overt developmental requirements for BMPR1a in many tissues, but indicated the LysMCre BMPR1a mouse model suited the objective of this study.

BMPs have also been found to impact myeloid cells when they are transformed into leukemia. In early stages of leukemia, BMPs are secreted by the surrounding microenvironment, but as disease progresses the leukemia stem cell population undertakes heightened BMP signaling (40). In our BMPR1a cKO mouse model, we observed variations in the immune microenvironment of tumor naïve and tumor bearing mice. Research in chronic myelogenous leukemia (CML) has uncovered BMPs as drivers of leukemia stem cell survival and expansion of myeloid progenitors to support disease progression (41). We observed that CD11b+/Ly6C+ cells in tumor naïve bone marrow and spleen tissues decreased upon loss of BMPR1a signaling, confirming the supporting role of BMPs in maintaining myeloid progenitor cell populations. T cell and B cell populations were not changed upon BMPR1a deletion in tumor naïve mice. Tumor bearing mice exhibited no changes in their CD11b+ myeloid, CD11b+/Ly6C+ monocyte and CD11b+/Ly6G+ neutrophil populations in cKO mice, but macrophages were significantly increased in the spleen and tumor. In the spleen of BMPR1a cKO mice, T cells were decreased while B cell increased. Tyrosine kinase inhibitor resistant CML patients exhibit higher BMP4 production and its receptor BMPR1b to form a CML promoting autocrine loop (42). Genetically inhibiting myeloid BMP signaling reduces tumor progression in our mouse model, confirming the requirement of BMPs in certain cancer contexts. Acute myeloid leukemia (AML) patients who express high BMP-4 and BMPR1a have higher relapse risk due to enhanced leukemia stemness (43). Our study supports the concept that BMP signaling in myeloid cells promotes undifferentiated phenotypes.

BMPs play a vital role in skeletal development and homeostasis of bone remodeling mediated by osteoblasts and osteoclasts. Crosstalk of BMPs mediate the balance between osteoblast driven bone mineralization and osteoclast bone resorption. Since osteoclasts originate from a myeloid lineage, LysMCre BMPR1a deletion will also impact osteoclast monocyte progenitors and may be included in the loss of undifferentiated myeloid cells in cKO tissues (44). Osteoclast progenitors have also been found to exhibit a CD11b+/Ly6C^high^ phenotype, possibly contributing to the changes observed in myeloid cell populations in this study (45). BMPR1a loss in osteoclasts promotes osteoblast mediated bone mineralization (46). In another BMPR1a mature osteoclast knockout study, osteoblastic bone formation increased, confirming that BMPR1a signaling from osteoclasts affects osteoblast function (47). Interestingly, when BMPR1a is deleted in a osteoblast specific knockout mouse model, bone mass was increased due to reduced osteoclastogenesis, signifying the importance of downstream BMP signaling by RANKL or sclerostin to regulate bone biology (48). The number of osteoclasts and mineralization rate decreased in the osteoblast BMPR1a knockout model, the bone formation rate also decreased despite increased bone mass (49). These studies highlight once again the context dependent and discrete usage of BMPs in bone development and disease.

BMP signaling in cancer associated myeloid cells is similarly complex, influencing macrophage polarization and subsequent cancer progression. BMP-2 expression has been described in a plethora of macrophage subtypes, with M1 macrophages secreting particularly high levels of BMP-2 (50, 51). BMP-2 is also involved in monocyte chemotaxis and cell adhesion, and prevents their differentiation into M2 macrophages, demonstrating its role in pro-inflammatory signaling (52). M2 macrophages stimulate mesenchymal stem cell proliferation and osteogenic differentiation through BMP-2 signaling (53). Conversely, in acute lymphoblastic leukemia BMP-4 is secreted to drive anti-inflammatory myeloid phenotypes, with dendritic cell immunosuppressive polarization, reduced M1 pro-inflammatory signature, and increased M2 macrophage generation (10). BMP-4 secreted from bladder cancer cell lines favored the polarization of monocytes and macrophages into the M2 macrophage phenotype (54). Similarly, in renal cell carcinoma BMP-6 production supports M2 macrophages and subsequent cancer progression (55). BMP-7 has also been shown to promote M2 polarization to promote anti-inflammatory activity (56, 57). Wound healing is enhanced by BMP-12 driving M2 polarization and effector function (58). Taken together these studies demonstrate a complex BMP ligand capacity to enforce a restricted macrophage inflammatory subset. In our study, the loss of BMP signaling in M2 polarized macrophages reduced the M2 pro-tumorigenic phenotype. This suggests that BMPR1a deletion in prostate tumor macrophages may inhibit the growth of tumors *in vivo*. Thus, targeting BMP signaling in macrophages may be a viable cancer therapy approach for reducing prostate cancer progression in patients.

In our previous breast cancer mouse model, treating mice with DMH1 reduced tumor progression and metastasis (15). This study also demonstrated that systemic BMP inhibition restricted M2 macrophage development in macrophages isolated from tumors, with reduced *Nos2, Il-10, Il-18*, and *Cox2* gene expression (15). In wild type monocytes from tumor naïve mice, DMH1 treatment resulted in reduced M2 gene expression by *Arg1, Il-10, Il-4, Mmp2, Mmp9*, and *Mmp13* (15). This suggests that BMPs are required for a unique myeloid and macrophage lineage that promotes cancer. Genetic and pharmacologic inhibition of BMP signaling is sufficient to alter the myeloid microenvironment of tumors as well as spleen and bone marrow compartments in this study. Overall, a shift toward a M1 phenotype was observed upon BMPR1a deletion in myeloid cells, yet gene expression of M1 and M2 markers were variable across Raw264.7, THP-1, and PODS cell lines. In cancer and many diseases, macrophages reflect a broader and more complex phenotype than simply M1 or M2 polarization (59). M1 and M2 signatures are no longer thought of as exclusive, rather they often coexist as a spectrum dependent upon cell type transcriptional responses (59, 60). A combination of markers were used in our experiments to better delineate macrophage polarization phenotypes, as individual genes are not sufficient to specify macrophage subsets (61). For example, *Il-6* is expressed in M1 and a subset of M2 macrophages, thus only in combination with other markers can *Il-6* expression help understand macrophage polarization (62). Aligning the expression of a M1 marker such as *TNF*α or a M2 marker such as *IL-6* with functional activity will help determine the signaling consequences in tumor associated macrophages. Beyond reducing the M2 phenotype of macrophages, the reduction in prostate tumor progression highlights a potential new paradigm to rewire the tumor microenvironment toward anti-tumor M1 macrophages.

Advanced prostate cancer patients face limited treatment options as their disease progresses under androgen-deprivation therapy and chemotherapy resistance. To alleviate tumor therapy resistance, the tumor microenvironment has become the target of basic and translational prostate cancer research (63). Myeloid cells are an important component of the tumor microenvironment and maintain signals in the stroma surrounding the tumor to either promote or inhibit tumor growth. Future studies into the role of BMPs in other pro-tumorigenic and anti-tumorigenic mechanisms such as phagocytosis can advance the field of therapeutic approaches for prostate cancer (64). Prostate cancer metastasizes most commonly to the bone, and induces tumor induced bone disease that results in extended suffering before patients succumb to the disease. Prostate cancer cells as well as the stroma of the bone marrow are supported by BMP signaling to drive bone metastases (65). A recent study showed that inhibition of BMP signaling improved bone health without increasing tumor growth in the bones of a multiple myeloma mouse model (66). Our findings support further investigation into how myeloid BMP drives tumor progression, and how to target BMP signaling in the metastatic tumor microenvironment.

## Data Availability Statement

The raw data supporting the conclusions of this article will be made available by the authors, without undue reservation, to any qualified researcher.

## Ethics Statement

The animal study was reviewed and approved by Vanderbilt IACUC, Vanderbilt University.

## Author Contributions

PO conceived and designed experiments. CI, MP, DS, SN, and PO performed experiments. CI and PO wrote and edited the manuscript.

### Conflict of Interest

The authors declare that the research was conducted in the absence of any commercial or financial relationships that could be construed as a potential conflict of interest.
