# Supplementary material for: Loss of Myeloid BMPR1a Alters Differentiation and Reduces Mouse Prostate Cancer Growth
Source: Front Oncol. 2020 Apr 7;10:357. doi: 10.3389/fonc.2020.00357 (PMC7154049; doi:10.3389/fonc.2020.00357)
Supplement: Supplementary file 1 [file Table_1.DOCX]

| **Supplemental Table 1. RT-PCR Primers** | | |  |
| --- | --- | --- | --- |
| Species | Target | Forward Primer | Reverse Primer |
| Mouse | *Id1* | CCTAGCTGTTCGCTGAAGGC | CTCCGACAGACCAAGTACCAC |
|  | *Tgfβ1* | CCACCTGCAAGACCATCGAC | CTGGCGAGCCTTAGTTTGGAC |
|  | *Mmp2* | ACCTGAACACTTTCTATGGCTG | CTTCCGCATGGTCTCGATG |
|  | *Mmp12* | CTGCTCCCATGAATGACAGTG | AGTTGCTTCTAGCCCAAAGAAC |
|  | *Nos2* | GTTCTCAGCCCAACAATACAAGA | GTGGACGGGTCGATGTCAC |
|  | *Cxcl10* | CCAAGTGCTGCCGTCATTTTC | GGCTCGCAGGGATGATTTCAA |
|  | *Tnfα* | CAGGCGGTGCCTATGTCTC | CGATCACCCCGAAGTTCAGTAG |
|  | *Il-1β* | GCAACTGTTCCTGAACTCAACT | ATCTTTTGGGGTCCGTCAACT |
|  | *Vegf164a* | CAGAAAATCACTGTGAGCCTTGTT | CTTGGCTTGTCACATCTGCAA |
|  | *Il-1rα* | GCTCATTGCTGGGTACTTACAA | CCAGACTTGGCACAAGACAGG |
|  | *Il-10* | GCTCTTACTGACTGGCATGAG | CGCAGCTCTAGGAGCATGTG |
|  | *Il-6* | TAGTCCTTCCTACCCCAATTTCC | TTGGTCCTTAGCCACTCCTTC |
|  | *Acvr1* | GTGGAAGATTACAAGCCACCA | GGGTCTGAGAACCATCTGTTAGG |
|  | *Bmpr1b* | CCCTCGGCCCAAGATCCTA | CAACAGGCATTCCAGAGTCATC |
|  | *Bmpr2* | TTGGGATAGGTGAGAGTCGAAT | TGTTTCACAAGATTGATGTCCCC |
| Human | *ID1* | CTGCTCTACGACATGAACGG | GAAGGTCCCTGATGTAGTCGAT |
|  | *TGFβ1* | CAATTCCTGGCGATACCTCAG | GCACAACTCCGGTGACATCAA |
|  | *MMP2* | TACAGGATCATTGGCTACACACC | GGTCACATCGCTCCAGACT |
|  | *MMP12* | GATCCAAAGGCCGTAATGTTCC | TGAATGCCACGTATGTCATCAG |
|  | *NOS2* | AGGGACAAGCCTACCCCTC | CTCATCTCCCGTCAGTTGGT |
|  | *CXCL10* | GTGGCATTCAAGGAGTACCTC | TGATGGCCTTCGATTCTGGATT |
|  | *ALOX15* | TGGAAGGACGGGTTAATTCTGA | GCGAAACCTCAAAGTCAACTCT |
|  | *IL-15* | TTTCAGTGCAGGGCTTCCTAA | GGGTGAACATCACTTTCCGTAT |
|  | *VEGFa* | AGGGCAGAATCATCACGAAGT | AGGGTCTCGATTGGATGGCA |
|  | *F13A1* | AGCTGGAGCTATGGTCAGTTT | TGACTTTGATGGGATTCCCTCT |
|  | *IL-10* | GACTTTAAGGGTTACCTGGGTTG | TCACATGCGCCTTGATGTCTG |
|  | *CCL13* | CTCAACGTCCCATCTACTTGC | TCTTCAGGGTGTGAGCTTTCC |

| **Supplemental Table 2. Fluorescent Antibodies from Biolegend** | | |
| --- | --- | --- |
| Antigen | Clone | Fluorophore |
| CD45 | 30-F11 | Qdot650 |
| CD11b | M1/70 | Pe-Cy5 |
| Ly6C | HK1.4 | Alexa Fluor 700 |
| Ly6G | 1A8 | APC-Cy7 |
| F4/80 | BM8 | APC |
|  |  |  |

| **Supplemental Table 3. CyTOF Antibodies from Fluidigm** | | |
| --- | --- | --- |
| Antigen | Clone | Channel |
| Ly6G/C (Gr1) | RB6-8C5 | 141Pr |
| CD11c | N418 | 142Nd |
| IL-5 | TRFK5 | 143Nd |
| IL-2 | JES6-5H4 | 144Nd |
| CD69 | H1.2F3 | 145Nd |
| F4/80 | BM8 | 146Nd |
| CD45 | 30-F11 | 147Sm |
| CD11b (MAC1) | M1/70 | 148Sm |
| CD19 | 6D5 | 149Sm |
| CD25 | 3C7 | 151Eu |
| CD3e | 145-2C11 | 152Sm |
| TER-119 | TER119 | 154Sm |
| IL-10 | JES5-16E3 | 158Gd |
| CD62L | MEL-14 | 160Gd |
| iNOS | CXNFT | 161Dy |
| TNFα | MP6-XT22 | 162Dy |
| IFNγ | XMG1.2 | 165Ho |
| IL-4 | 11B11 | 166Er |
| IL-6 | MP5-20F3 | 167Er |
| CD8a | 53-6.7 | 168Yb |
| TCRβ | H57-597 | 169Tm |
| NK1.1 | PK136 | 170Er |
| CD44 | IM7 | 171Yb |
| CD4 | RM4-5 | 172Yb |
| IL-17A | TC11-18H10.1 | 174Yb |
| B220 | RA3-6B2 | 176Yb |
