# Supplementary material for: Loss of Myeloid BMPR1a Alters Differentiation and Reduces Mouse Prostate Cancer Growth
Source: Front Oncol. 2020 Apr 7;10:357. doi: 10.3389/fonc.2020.00357 (PMC7154049; doi:10.3389/fonc.2020.00357)
Supplement: Supplementary file 2 [file Data_Sheet_1.PDF]

Suppl Fig 1 Ihle et al.

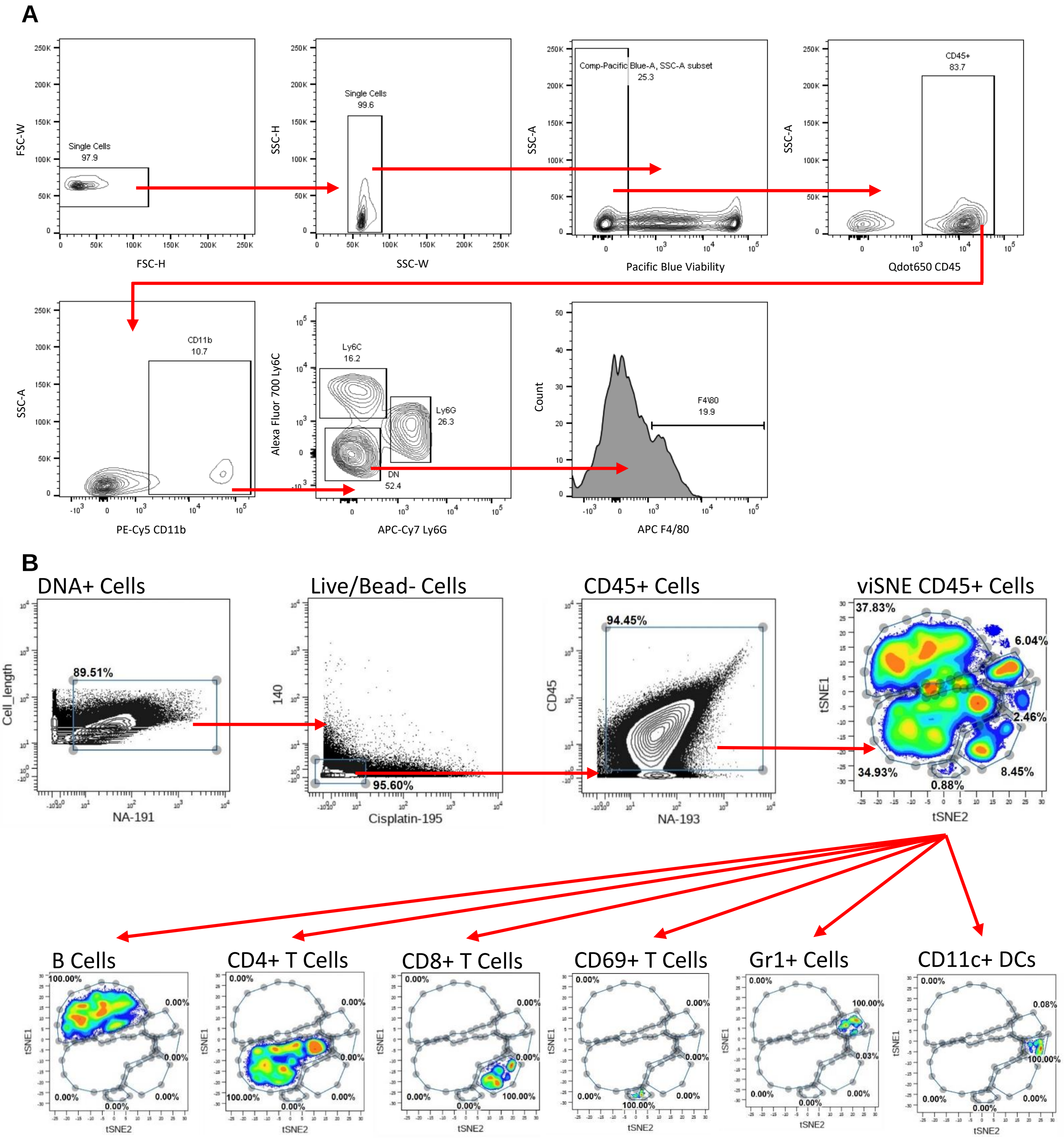

Supplemental Figure 1. Gating Strategies for Flow Cytometry and CyTOF. (A) Flow cytometry analysis performed in FlowJo. Gating strategy for assessment of mouse spleen shown, tumor and bone marrow gating followed the same strategy for analysis. **(B)** CyTOF analysis performed in Cytobank. Cells gated off of a DNA+ gate, followed by a Live Cell gate then a CD45+ gate to isolate immune cells. viSNE clustering implemented on all CD45+ cells and included all staining markers, with gates drawn around immune cell populations based on positive staining for each immune marker from the viSNE clusters. Immune populations identified for CD19+/CD20+ B cells, CD4+ T cells, CD8+ T cells, CD69+ T cells, Gr1+ cells, CD11b+ cells, and CD11c+ dendritic cells shown on lowest panel for positive marker staining from viSNE. Gating strategy for assessment of mouse spleen shown, tumor and bone marrow gating followed a similar strategy for analysis.

Suppl Fig 2 Ihle et al.

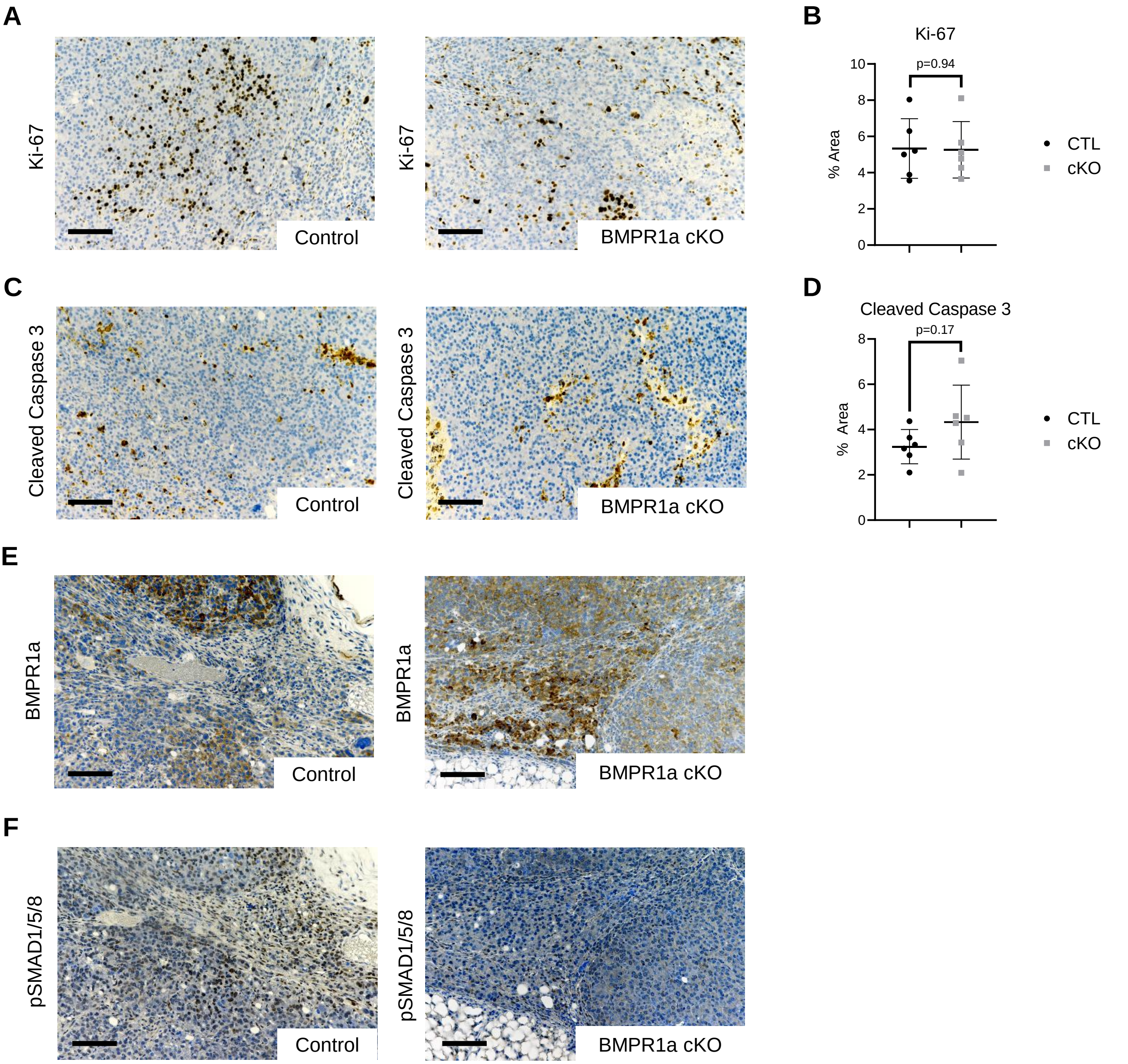

Supplemental Figure 2. Tumor Proliferation, Apoptosis and BMPR1a Expression and Signaling in BMPR1a cKO Mice. Mouse tumors from control (CTL) and LysMCre BMPR1a knockout (cKO) male mice with flank MyC-CaP tumors were harvested then stained by IHC. Staining intensities were averaged for both flank tumors files from each mouse by ImageJ. **(A)** Representative image of IHC staining for Ki-67 at day 29. Scale bars indicate 100µm. **(B)** Quantitation of Ki-67 staining for all CTL and cKO tumors. Mean graphed with SD, p-value calculated by Student t-test. **(C)** Representative image of IHC staining for cleaved caspase 3 at day 29. Scale bars indicate 50µm. **(D)** Quantitation of cleaved caspase 3 staining for all CTL and cKO tumors. Mean graphed with SD, p-value calculated by Student t-test. **(E)** Representative image of IHC staining for BMPR1a at day 29. Scale bars indicate 100µm. **(F)** Representative image of IHC staining for pSMAD1/5/8 at day 29. Scale bars indicate 100µm.

Suppl Fig 3 Ihle et al.

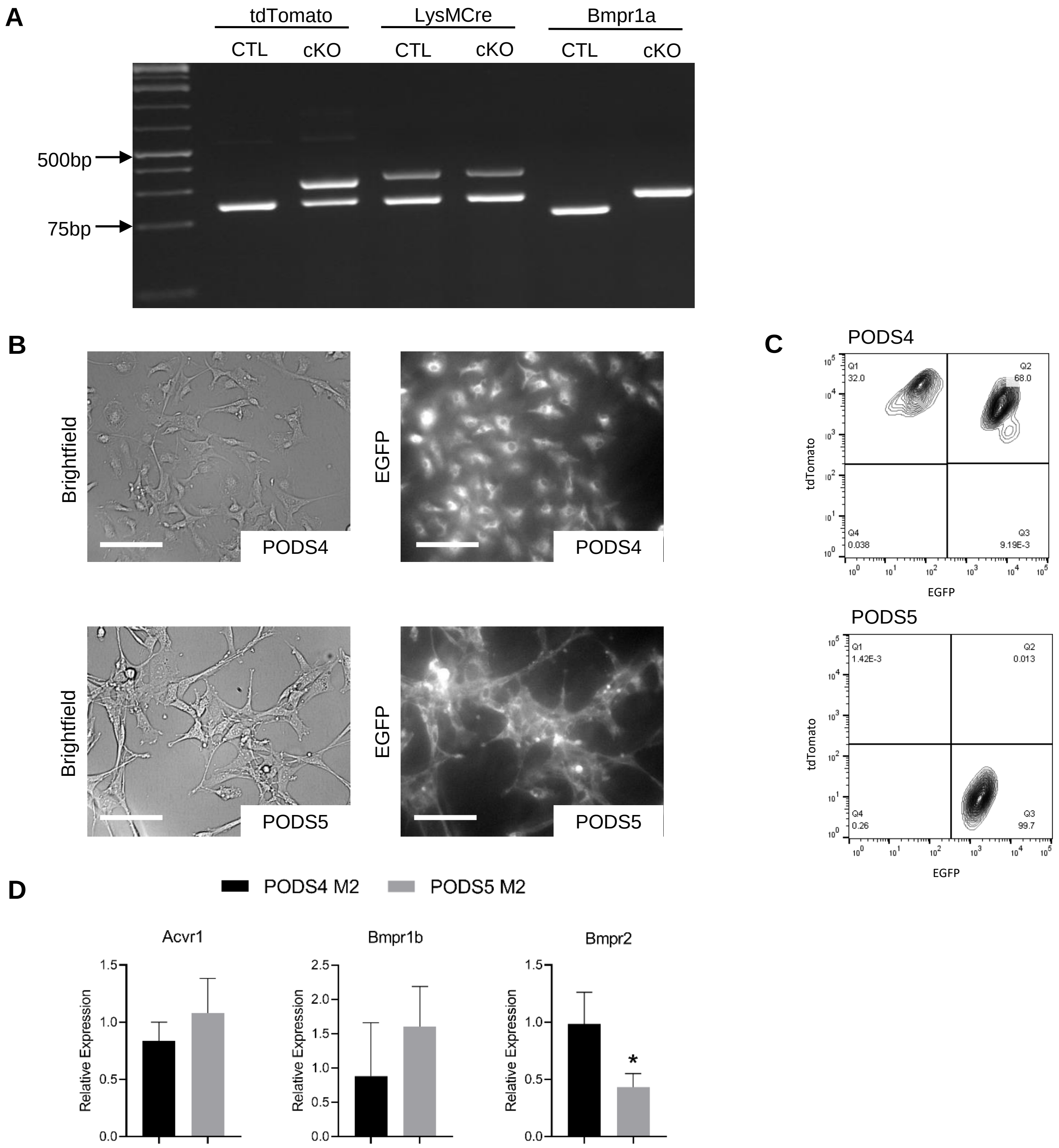

Supplemental Figure 3. Generation of Bone Marrow Derived BMPR1a Control PODS4 and BMPR1a cKO PODS5 Primary Cell Lines. Mouse bone marrow from control (CTL) and LysMCre BMPR1a knockout (cKO) male mice were harvested then cultured to generate PODS4 and PODS5 cell lines. **(A)** Genotyping gel for CTL (PODS4) and cKO (PODS5) cell lines. Band sizes include tdTomato mutant band at 196bp, wild type tdTomato band at 297bp, LysMCre specific band at 320bp, Cre control myogenin band at 250bp, Bmpr1a mutant band at 200bp, and wild type Bmpr1a band at 75bp. **(B)** Brightfield and EGFP imaging of PODS4 and PODS5 cell lines. Scale bars indicate 100 µm. **(C)** Flow cytometry sort of PODS4 and PODS5 based on EGFP and tdTomato expression. **(D)** Gene expression of BMP receptor expression in PODS4 and PODS5 cell lines polarized into M2 macrophages in triplicate. Mean graphed with SD, * indicates statistical significance p≤0.05 and ** indicates p≤0.01 by Student t-test.
